# The clinical effect of a new infant formula in term infants with constipation: a double-blind, randomized cross-over trial

**DOI:** 10.1186/1475-2891-6-8

**Published:** 2007-04-11

**Authors:** Marloes EJ Bongers, Fleur de Lorijn, Johannes B Reitsma, Michael Groeneweg, Jan AJM Taminiau, Marc A Benninga

**Affiliations:** 1 Department of Pediatric Gastroenterology and Nutrition, Emma Children's Hospital, Academic Medical Centre, Amsterdam, The Netherlands; 2 Department of Clinical Epidemiology and Biostatistics, Academic Medical Centre, Amsterdam, The Netherlands; 3 Department of Pediatrics, Medical Centre Rijnmond-Zuid, Rotterdam, The Netherlands

## Abstract

**Background:**

Nutrilon Omneo (new formula; NF) contains high concentration of *sn-2 *palmitic acid, a mixture of prebiotic oligosaccharides and partially hydrolyzed whey protein. It is hypothesized that NF positively affects stool characteristics in constipated infants.

**Methods:**

Thirty-eight constipated infants, aged 3–20 weeks, were included and randomized to NF (n = 20) or a standard formula (SF; n = 18) in period 1 and crossed-over after 3 weeks to treatment period 2. Constipation was defined by at least one of the following symptoms: 1) defecation frequency < 3/week; 2) painful defecation; 3) abdominal or rectal palpable mass.

**Results:**

Period 1 was completed by 35 infants. A significant increase in defecation frequency (NF: 3.5 pre versus 5.6/week post treatment; SF 3.6 pre versus 4.9/week post treatment) was found in both groups, but was not significantly different between the two formulas (p = 0.36). Improvement of hard stool consistency to soft stool consistency was found more often with NF than SF, but did not reach statistical significance (90% versus 50%; RR, 1.8; 95% CI, 0.9–3.5; p = 0.14). No difference was found in painful defecation or the presence of an abdominal or rectal mass between the two groups. Twenty-four infants completed period 2. Only stool consistency was significantly different between the two formulas (17% had soft stools on NF and hard stools on SF; no infants had soft stools on SF and hard stools on NF, McNemar test p = 0.046).

**Conclusion:**

The addition of a high concentration *sn-2 *palmitic acid, prebiotic oligosaccharides and partially hydrolyzed whey protein resulted in a strong tendency of softer stools in constipated infants, but not in a difference in defecation frequency. Formula transition to NF may be considered as treatment in constipated infants with hard stools.

## Background

Between 16–40% of the infants with constipation experience symptoms before the age of six months [1-3]. In approximately 90% of infants no specific organic cause can be found [4]. It is well established that the bowel pattern in infants is influenced by the type of feeding in the first months after birth. Constipation is more commonly found in formula-fed infants, who have a greater tendency to produce hard stools compared to breast-fed infants [5]. Differences in the composition between breast- and formula feeding may explain this finding.

The structure of lipids differs between human milk and infant formulas. In both human milk and infant formulas palmitic acid is the predominant saturated fatty acid. In human milk 70–85% of palmitic acid is positioned at the sn-2 position of the triacylglycerol molecule, whereas in regular infant formulas 88–94% of palmitic acid is found at the sn-1 and sn-3 position [6-10]. Lipolysis of triacylglycerol by pancreatic lipase occurs predominantly at the sn-1 and sn-3 positions, yielding free fatty acids and a 2-monoacylglycerol [11,12]. Subsequently, free palmitic acid may form insoluble calcium fatty acid soaps which are excreted via the feces, resulting in firmer stools. Stool hardness has been positively associated with the presence of calcium fatty acid soaps in the stools [5]. In human milk however, palmitic acid esterified at the sn-2 position of the triacylglycerol molecule is well absorbed as 2-monopalmitin, since it readily forms mixed micelles with bile acids [11,13-15].

Human milk is further known to be a rich source of oligosaccharides [16]. These oligosaccharides resist digestion in the small intestine and thus reach the colon unaltered, where they serve as prebiotics [17]. They act as growth substrate for bifidobacteria, which are thought to have beneficial effects on the host's health by supporting the gut barrier, stimulating normal intestinal function, and strengthening the immune system [18-20]. In addition, due to their non-digestibility, they may be considered to be a form of soluble fibres and contribute to the softer stools produced by breast-fed infants [17,21].

Based on these findings, the concept of adding modified triacylglycerol and prebiotic oligosaccharides to infant formulas has arisen. A new infant formula (NF; Nutrilon Omneo, Nutricia Nederland BV, Zoetermeer, the Netherlands) was developed which contains modified vegetable oil with a high proportion (41%) of palmitic acid at the *sn-2 *position, a mixture of prebiotic oligosaccharides, partially hydrolyzed whey protein and a reduced lactose content. The oligosaccharides mixture consists of 90% short-chain galacto-oligosaccharides (GOS) and 10% long-chain fructo-oligosaccharides (lcFOS), 0.8 g/100 ml, and resembles human milk oligosaccharides with respect to its molecular weight distribution and high galactose content [22]. The effect of NF on stool frequency and consistency has been assessed in one study in healthy term infants [23]. Infants receiving NF were found to produce softer stools than those fed a regular infant formula. We hypothesized that this NF will also have a positive effect stool characteristics in constipated infants.

## Methods

### Patients

This study was conducted in the academic medical hospital in Amsterdam and 5 non-academic hospitals in the Netherlands. Eligible for the study were otherwise healthy, term infants with constipation, between 3 – 20 weeks of age, who received at least 2 bottles of milk-based formula per day. Constipation was defined as the presence of at least one of the following symptoms: 1) frequency of defecation < 3/week; 2) painful defecation (crying); 3) abdominal or rectal palpable mass [3]. Children with Hirschsprung's disease, spinal or anal anomalies, previous colonic surgery, metabolic, cerebral and renal abnormalities were excluded. Also children who were treated with laxatives at enrollment were excluded. The medical ethics committees of the participated hospitals approved the research protocol. All parents gave written informed consent.

### Medical history and physical examination

At enrollment, clinical history, dietary history, obstetrical data and anthropometry were recorded. The infants were randomized by a computer program to either NF or SF in period 1 and crossed-over after 3 weeks to treatment period 2. In order to mimic the taste of Nutrilon Omneo, the whey-based control formula was partly mixed with a formula based on hydrolyzed whey protein (mixture of 75% Nutrilon 1 and 25% Aptamil HA l). Further details on the composition of the study formulas are given in Table 1. Formula cans were labeled with codes to mask identity of the study feedings. Neither the parent nor the physicians were aware of the composition of the formula until the entire study was completed.

**Table 1:** Composition of the study formulas

Nutrients per 100 ml	SF *	NF
Energy (kcal)	67	70
Protein (g)	1.5	1.7
Casein	0.5	-
Intact whey protein	0.6	-
Whey protein hydrolysate	0.4	1.7
Fat (triglycerides) (g)	3.5	3.3
Palmitic acid	0.6	0.6
- at the *sn-2 *position (%)	11.5	41.0
Linoleic acid	0.4	0.4
α-linolenic acid	0.07	0.08
Carbohydrates (g)	7.3	8.4
Lactose	7.2	2.9
Maltodextrin	-	4.0
Starch	-	1.5
Fibre (g)	-	0.8
Oligosaccharides (90% GOS, 10% lcFOS)	-	0.8
Minerals and trace elements (mg)		
Calcium	53	53
Phosphorus	29	29
Sodium	22	23
Potassium	69	82
Chloride	42	44
Iron	0.5	0.5
Zinc	0.5	0.5

* mixture of 75% Nutrilon 1 and 25% Aptamil HA 1

During both periods parents were asked to daily record in a diary details on formula intake, formula tolerance (vomiting, flatulence, colics, rash), passage of stools and stool consistency compared to four validated photographs of runny, mushy soft, formed soft and hard stools [21]. After each intervention period, an out-patient clinic visit for evaluation of data was done. During these visits, anthropometric assessments and abdominal and rectal examination were performed.

### Efficacy parameters

In this study it was hypothesized that the use of NF would alleviate the symptoms of constipation. The following primary efficacy parameters were assessed: 1) defecation frequency > 3/week; 2) normalization of stool consistency; 3) no more painful defecation; 4) absence of abdominal or rectal palpable mass at physical examination. Secondary outcome measures were formula tolerance and weight gain.

### Statistical analysis

Prior to the start of the study, sample size, based on a cross-over design, was calculated to allow detection of a 30% difference in improvement between NF and SF. Under the assumption of a significance level of 0.05 with a power of 0.80, and 2-sided hypothesis testing, a minimal sample size of 34 with 17 children in each group was determined.

**Figure 1:**
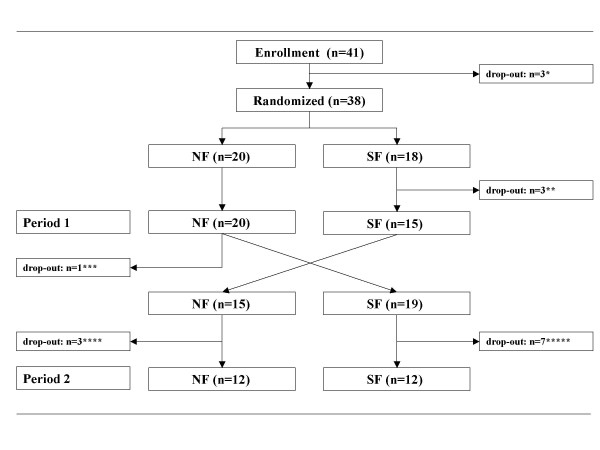
Trial profile * No contact after intake. ** In period 1 three SF patients dropped out; 2 patients stopped because of severe constipation; one patient switched to hypoallergenic feeding, because of suspected cow's milk protein allergy. *** Parents of 1 patient decided that they did not want to cross-over because she was free of symptoms and they started openly with NF instead. **** Three patients dropped out after switching to NF; 2 patients stopped after less than 1 week because of recurrence of constipation symptoms. 1 patient was lost to follow-up. ***** Seven patients dropped out after switching to SF; 6 patients stopped after one week because of recurrence of constipation symptoms. 1 patient was lost to follow-up.

Descriptive statistical measures were calculated for baseline characteristics. Our cross-over study was hampered by the drop-out of a substantial number of children after they finished period 1 and before or during the second period of treatment (Figure 1). Only 24 children (63%) completed the cross-over study. It is highly likely that drop-out occurred not at random, but was related to clinical outcome (for instance early termination in period 2 because of insufficient response). A cross-over analysis of the completers within this study could therefore lead to biased results. Therefore we restricted our main analysis to the first period. Essentially, this reduces our trial to a simple, two-group parallel trial, but we rather sacrifice loss-of-power than introducing bias. Comparisons between the two treatment groups after period 1 were performed using ANCOVA in case of continuous outcomes and χ^2 ^tests for categorical endpoints. However, we do present the results of the patients that finished the cross-over trial to evaluate whether the results are in the same line as the first period results. The subgroup analysis was performed using either a paired sample *t *test or the McNemar test for paired observations. Differences were considered statistically significant when the P-value was less than 0.05.

## Results

### Patient characteristics

Between April 2002 and January 2004, 41 constipated infants were enrolled in the study. Directly after inclusion 3 infants dropped out for unknown reasons, because parents did not show up at the next outpatient clinic visit. Thus, 38 patients were randomized and received either SF (n = 18) or NF (n = 20). Figure 1 shows a scheme of the trial profile. A total of 35 infants completed the first period of 3 weeks. Only 24 patients completed the full cross-over study. The various reasons for withdrawal of the study are described in the legends of Figure 1. Data analysis was based on the group of 35 patients that completed period 1 and a subgroup analysis of 24 patients who completed the cross-over.

The median age at enrollment of the 38 infants (19 male) was 1.7 months, whereas the median age at onset of constipation was 2 weeks. The mean defecation frequency was 3,5/week and 45% of all infants had a defecation frequency of less than 3 times per week. Furthermore, the main symptoms of constipation were hard stool consistency and painful defecation, occurring in 61% and 82% of infants respectively. Baseline characteristics of the infants according to randomization to SF or NF are described in Table 2.

**Table 2:** Baseline characteristics of infants with functional constipation by randomized group

Characteristics	SF (n = 18)	NF (n = 20)	P- value
Number of boys (%)	11 (61)	8(40)	0.19
Age at intake (in months)			
median	1.8	1.7	0.80
min-max	1.1–5.0	0.7–3.7	
Age of onset (in weeks)			
median	2.0	2.0	0.45
min-max	0–20	0–6	
Defecation frequency			
N/week (mean ± SD)	3.6 ± 1.8	3.5 ± 2.6	0.64
< 3 times/week	39%	50%	0.49
Hard stool consistency	72%	50%	0.16
Painful defecation	89%	75%	0.27
Abdominal scybalus	44%	20%	0.13
Rectal scybalus	29%	21%	0.51
Meconium passage < 48 hours	100%	90%	0.32
Positive family history	61%	58%	0.84

### Clinical efficacy after period 1

Compared to baseline, a significant increase in mean defecation frequency/week was found after 3 weeks from 3.5/week to 5.3/week (difference between means, 1.8; 95% CI: 0.81 – 2.78; p = 0.001). Mean defecation frequency/week increased from 3.5/week pre to 5.6/week post treatment with NF compared to 3.6/week pre to 4.9/week post treatment with SF, but the increase in frequency was not significantly different between the groups (difference between means, 0.7; 95% CI, -0.8 – 2.3; p = 0.36, ANCOVA) (Table 3). Improvement of hard stool consistency at intake to soft stool consistency after 3 weeks intervention was found more often with NF than SF, but did not reach statistical significance (90% versus 50%; RR, 1.8; 95% CI, 0.9 – 3.5; p = 0.14). Furthermore, painful defecation diminished in both groups; NF: 75% pre and 65% post treatment and SF: 89% pre and 67% post treatment. Also a decrease in the presence of an abdominal and/or rectal mass was found; NF: 35% pre and 10% post treatment and SF: 44% pre and 5.6% post treatment. However, no significant differences in both of these patient characteristics were found between the two feeding groups (Table 3).

**Table 3:** Clinical efficacy of SF versus NF after period 1

	SF (n = 15)	NF (n = 20)	Difference of means (95% CI)*	RR (95% CI)**	P-value
Defecation frequency (mean ± SD)	4.9 ± 2.5	5.6 ± 2.8	0.7 (-0.8–2.3)		0.36
Improvement of hard to soft stools (n)	50% (5/10)	90% (9/10)		1.8 (0.9–3.5)	0.14
No painful defecation (n)	33% (5/15)	35% (7/20)		1.0 (0.4–2.7)	0.92
No abdominal or rectal mass (n)	93% (14/15)	90% (18/20)		1.0 (0.8–1.2)	0.73

* ANCOVA; ** χ^2 ^tests

### Clinical efficacy after cross-over (period 1 and 2)

Only 24 infants completed the cross-over study. In these infants, the defecation frequency was comparable for NF and SF with a mean frequency of 5.5/week vs. 5.9/week (Difference of means, - 0.5; 95% CI, -1,6 – 0,6; p = 0.38), respectively. The frequency of soft stools was significantly higher in the NF period, with 17% (n = 4) of infants having soft stools when receiving NF but hard stools with SF, compared to no infant with soft stools when receiving SF and hard stools with NF (McNemar test, p = 0.046). Painful defecation and the presence of abdominal or rectal mass were not significantly different between the periods on NF and SF.

### Safety

Throughout the study there were no serious adverse effects in either group. Both formulas were well tolerated. Weight gain was similar in both feeding groups. In period 1 NF fed-infants gained 29.7 grams/day whereas weight gain in infants fed SF was 32.2 grams/day (difference of means, -2.6; 95% CI, -11.7 – 6.6; p = 0.57). In the subgroup that completed the cross-over phase of the study, growth was not significantly different between the periods on NF and on SF; 28.2 grams/day versus 33.5 grams/day respectively (difference of means, -5.4; 95% CI, -13.0 – 2.3; p = 0.16).

## Discussion

To our knowledge this is the first double-blind cross-over study evaluating the effect of Nutrilon Omneo (NF) on stool characteristics in infants with constipation. Our data show that constipated infants with hard stools fed NF, containing a high proportion of *sn*-2 palmitic acid and a mixture of prebiotic oligosaccharides, improved more often to softer stools compared to whey-based SF-fed infants. Defecation frequency increased significantly compared to baseline in both groups, but was not different between the NF-fed and SF-fed infants. The formula was well tolerated and growth rates were similar on both formulas.

Previous studies in healthy infants revealed a wide variability in defecation frequency depending on age and the type of feeding [21,24,25]. Fontana et al. showed a decline in defecation frequency from an average of 3 stools per day in the first month of life to 1.4 per day at 3 years of age [24]. Furthermore, breast-fed infants have a defecation frequency twice as high as formula-fed infants in the first 12 weeks of life [21]. In this study in infants with constipation, the mean defecation frequency at enrollment was 3.5 times per week. A total of 45% of all infants presented with a defecation frequency of < 3 times/week. Two recent follow-up studies in constipated infants found different defecation frequencies at enrollment of 6.5 times per week and 2 times per week, respectively [26,27]. This disparity is most likely explained by the difference in patient populations. The study by Loening-Baucke et al. and our study were conducted in infants referred to general pediatric out-patient clinics. In the study by Van den Berg et al. only infants with severe functional constipation that required referral to a specialized clinic to rule out Hirschsprung's disease were included. The difference in age distribution between the studies may also have contributed to the difference in outcome, since we included younger infants up to 5 months of age, but the other two studies included children up to a maximum of 2 years of age [26,27].

In comparison to these recent follow-up studies in constipated infants, we found a higher percentage of infants with painful defecation (82%). Hard stool consistency in more than half of the infants just partially explains this finding, since the presence of painful defecation did not improve in accordance with a softer stool consistency. Loening-Baucke et al. reported hard stool consistency in 93% of all constipated children and painful defecation in only 41%. Van de Berg et al. did not report on stool consistency, but painful defecation was present in 49% of the infants. Differences may be explained by the fact that painful defecation at this young age is a very subjective measurement and difficult to objectify by a parent on a daily basis. The fact that this study was a multi-centre trial may also have lead to an inter-observer difference in the registration of changes in painful defecation.

In our analysis infants receiving NF showed more improvement of hard stools to soft stools than those fed SF, although significance was only reached in infants completing the cross-over study. This result is in line with the findings of two previous studies demonstrating softer stools in healthy term infants fed high *sn*-2 palmitate formula [9,15]. This effect is attributed to a reduced fecal excretion of calcium-fatty acid soaps [5,28]. Another explanation for the difference in stool consistency is most likely the addition of a prebiotic mixture of GOS/lcFOS in the Nutrilon Omneo formula. Earlier studies in healthy infants have shown that supplementation of an infant formula with this mixture resulted in an increased number of fecal *Bifidobacteria *and softer stool consistency [23,29,30]. Moro et al. found that this effect was dose-dependent and softer stools were comparable to stools of breast-fed infants at a level of 0.8 g/100 ml of GOS/FOS mixture [30]. Similar to our results, Schmelzle et al. showed softer stools, but no statistical difference in defecation frequency in healthy term infants fed NF [23]. This positive effect on stool consistency is of clinical importance in our patient population, as a majority of infants (61%) presented with hard stool consistency as main symptom of their constipation.

Additionally, previous studies have shown that formulas containing hydrolyzed protein can produce softer stools [31]. Therefore, the presence of hydrolyzed whey protein may also have contributed to the stool softening effect of NF. In this respect, the addition of some hydrolyzed protein (about 25% of total protein) to SF may explain the observed increase of defecation frequency on both formulas. However, the observed increase in defecation frequency was stronger in infants fed NF than those fed SF, but no significant difference was found.

A limitation of this study is that more than one third of the constipated infants enrolled did not complete the study protocol. Due to this high drop-out rate, no cross-over data was available from these children resulting in loss of power in our analysis. Therefore our findings on defecation frequency and stool consistency need to be confirmed in a larger clinical trial in order to gain more insight into the effects of the composition of this NF.

This drop-out rate is of further concern because it may potentially introduce a bias. However, besides a significantly lower age at enrollment in the drop-out group, baseline characteristics showed no difference between the infants who dropped out and the remaining infants. There was a relationship between the drop-out rate and the feeding type, since the most important reasons for drop-out were: 1) a significant improvement after the first treatment period on NF, or 2) the recurrence of symptoms of constipation after the switch to SF in the second period. Understandably, parents refused to continue the study when their infant had improved or when symptoms of constipation recurred. In general, parents of sick children have emotional and ethical problems to accept the risk of recurrence of the initial symptoms. The latter is probably the reason for the lack of scientific data evaluating the effect of infant formulas or oral laxatives in infants with constipation.

In conclusion, this study demonstrates that the use of an infant formula with a high proportion of *sn*-2 palmitate, a mixture of prebiotic galacto-oligosaccharides and fructo-oligosaccharides and partially hydrolyzed whey protein may lead to softer stools in constipated infants. Thus, constipated infants who present with hard stools may benefit from a change form SF to this NF as a first treatment step, but larger randomized clinical trials on the efficacy of this new formula are needed.

## Competing interests

This study was supported by a grant of Nutricia Nederland BV, Zoetermeer, The Netherlands.

## Authors' contributions

FdL contributed substantial to conception and design of the study and collection of the data. MIG contributed to the acquisition of data and has been involved in revising the manuscript. JBR contributed significantly to the statistical analysis. MEB carried out the statistical analysis and wrote the manuscript. JAT helped to critically revise the manuscript. MAB supervised the design and coordination of the study and helped to draft and revise the manuscript. All authors read and approved the final manuscript.
